# MicroRNAs in Cerebral Ischemia

**DOI:** 10.1155/2013/276540

**Published:** 2013-03-06

**Authors:** Yang Wang, Yongting Wang, Guo-Yuan Yang

**Affiliations:** ^1^Department of Neurology, Ruijin Hospital, Shanghai Jiao Tong University School of Medicine, Shanghai 200025, China; ^2^Neuroscience and Neuroengineering Research Center, Med-X Research Institute and School of Biomedical Engineering, Shanghai Jiao Tong University, Shanghai 200030, China

## Abstract

The risk of ischemic stroke increases substantially with age, making it the third leading cause of death and the leading cause of long-term disability in the world. Numerous studies demonstrated that genes, RNAs, and proteins are involved in the occurrence and development of stroke. Current studies found that microRNAs (miRNAs or miRs) are also closely related to the pathological process of stroke. miRNAs are a group of short, noncoding RNA molecules playing important role in posttranscriptional regulation of gene expression and they have emerged as regulators of ischemic preconditioning and ischemic postconditioning. Here we give an overview of the expression and function of miRNAs in the brain, miRNAs as biomarkers during cerebral ischemia, and clinical applications and limitations of miRNAs. Future prospects of miRNAs are also discussed.

## 1. Introduction

miRNAs are approximately 20-nucleotide, single-stranded RNA molecules that target mRNA through partial complementarity and they can regulate gene expression through inhibition of translation or transcript degradation [1]. It is now predicted that 40% to 50% of mammalian mRNAs could be regulated at the translational level by miRNAs [2]. In mammals, specific miRNAs are known to control processes including development, neuronal cell fate, apoptosis, proliferation, adipocyte differentiation, hematopoiesis, and exocytosis as well as in diseases [3–5] and possibly neuronal disorders [6]. miRNA expression has been detected in stroke [2, 7], Alzheimer's disease [8], Parkinson's disease [9], Down's syndrome [10], and schizophrenia [11]. These miRNAs expression profiles may be as diagnostically useful as mRNA expression profiles [12].

In the nucleus, miRNAs are transcribed as hairpin clusters of primary miRNAs (pri-miRNAs; 5′-capped polyadenylated transcripts), which is converted to 70-nt stem loop structures (pre-miRNAs) by Drosha (a type-III RNase) in association with a cofactor Pasha (aka DiGeorge syndrome critical region gene 8) [13]. pre-miRNAs are transported from nucleus to cytosol by exportin-5 and acted on by another type-III RNase known as Dicer that deletes the terminal loop of pre-miRNAs to form mature miRNAs [14]. 

## 2. miRNA Expression and Its Functions in the Brain

miRNAs serve important roles in the development and function of the brain [15–19]. Studies support that tissue-specific miRNAs contribute to establish and maintain protein expression profiles underlying distinct cellular phenotypes. The discovery of seven brain-specific miRNAs (miR-9, miR-124a, miR-124b, miR-135, miR-153, miR-183, and miR-219) in mouse and human differentiating neurons implicated these miRNAs as effectors in mammalian neuronal processes [20]. Further studies showed that expression levels of the brain-specific miR-124 are 100 times higher in mouse central nervous system than in other organs, whereas levels of muscle-specific miR-1 are 100 to 1000 times lower in mouse central nervous system than in heart and skeletal muscles [21]. Transfection of brain-specific miR-124 into HeLa cells shifted the expression profile toward that of the brain's, whereas transfection of the heart and skeletal muscle-specific miR-1 into HeLa cells shifted the expression profile toward that of the muscle's [22]. Among neural-derived cells, integrated mRNA-miRNA functional analyses of mature neurons (MNs), neural progenitor cells (NPCs), and neuroblastoma cells (NBCs) revealed that several very highly expressed genes (e.g., Robo1, Nrp1, Epha3, Unc5c, Dcc, Pak3, and Limk4) and a few underexpressed miRNAs (e.g., miR-152, miR-146b, and miR-339-5p) in MNs are associated with one important cellular process-axon guidance; some very highly expressed mitogenic pathway genes (e.g., Map2k1, Igf1r, Rara, and Runx1) and underexpressed miRNAs (e.g., miR-370, miR-9, and miR-672) in NBCs are associated with cancer pathways [23].

### 2.1. The Function of miRNAs in Cerebral Ischemia

Several reports have demonstrated the effects of specific miRNAs in neuronal differentiation, neurogenesis, neural cell specification, and neurodevelopmental function [6, 24]. In stroke etiology, miRNAs have distinct expression patterns that modulate pathogenic processes, including atherosclerosis (miR-21 and miR-126), hyperlipidemia (miR-33 and miR-125a-5p), hypertension (miR-155), and plaque rupture (miR-222 and miR-210) [25]. miRNA profiling (screening) was performed on rat brains subjected to middle cerebral artery occlusion (MCAO) and reperfusion for 24 or 48 hours. They identified the expression of 114 miRNAs in ischemic brain samples. Among them, 106 and 82 transcripts were detected in the 24-hour and 48-hour reperfusion brain samples, respectively [2]. To understand miRNAs' functional significance in ischemic pathophysiology, Dharap et al. reported the level of miRNAs in adult rat brain as a function of reperfusion time after transient MCAO [7]. Of the 238 miRNAs evaluated, 8 showed increased expressions and 12 showed decreased ones at least at 4 out of 5 reperfusion time points studied between 3 hours and 3 days compared with sham [7]. The differentially expressed miRNAs and their protein kinase c-(PKC) isoform specific gene network in mouse brain after HPC (hypoxic pre-conditioning) and 6 h MCAO are determined [26]. Moreover, anti-miR-320a could bring about a reduction of infarct volume in cerebral ischemia with a concomitant increase in aquaporins-1 and 4 mRNA and protein expression [27]. Tan and colleagues carried out miRNA profiling from peripheral blood of young stroke patients aged 18–49 years, and identified characteristic patterns in ischemic stroke [28]. 

### 2.2. Neuroprotection

miR-497 promoted ischemic neuronal death by repressing expression of Bcl-2 and Bcl-w, supporting the role of apoptosis in the pathogenesis of ischemic brain injury [29]. Knockdown of cerebral miR-497 in mice attenuated brain infarction, protected neuron, and improved neurological outcome after focal ischemia [29]. In rats subjected to transient cerebral ischemia, the brain-specific miR-134 and miR-124, involved in brain and neural tube development, respectively, are upregulated [2, 24, 30]. This process may be related to regeneration during the rest 24 hours of reperfusion in the injured brain cells. Anti-miR-1 treatment, as late as 4 hours following ischemia, significantly reduced cortical infarct volume in adult female rats, while anti-Let7 robustly reduced both cortical and striatal infarcts, and preserved sensorimotor function and interhemispheric neural integration. Antagomirs to miR-1 and Let7f, with consensus binding sites in the 3 UTRs of multiple IGF signaling pathway components confer neuroprotection, while antagomir to a brain-specific miRNA not associated with IGF signaling, was not neuroprotective [31]. Moreover, miR-34a was significantly upregulated at 1, 7, and 14 days after status epilepticus and at 2 months after temporal lobe epilepsy. Experiments with the miR-34a antagomir revealed that targeting miR-34a led to an inhibition of activated caspase-3 protein expression, which may contribute to increased neuronal survival and reduced neuronal death or apoptosis [32]. Besides, in astrocyte, miR-181 regulation of Bcl-2 and Mcl-1 contributes to mitochondrial dysfunction observed with in vitro ischemic injury, in this case glucose deprivation [33]. Increased miR-181a exacerbated injury both in vitro and in the mouse ischemia model [34]. 

### 2.3. Angiogenesis

miR-126 is recognized as the most important miRNA for maintaining vascular integrity during ongoing angiogenesis, as it targets SPRED1 and PIK3R2, two negative regulators of VEGFs signaling [35]. Growth factors increase the expression of the proangiogenic miR-130a and miR-296 in endothelia cells [36]. miR-130a stimulates angiogenesis by inhibiting GAX and HOXA5, while, miR-296 acts through the inhibition of hepatocyte growth factor-(HGF-) regulated tyrosine kinase [36]. miR-210 is induced by hypoxia in endothelial cells [37]. miR-210 overexpression enhances the formation of capillary-like structures and VEGF-driven migration of normoxic endothelial cells, whereas inhibition of miR-210 decreases tube formation and migration [37]. The modulation of endothelial cell responses to hypoxia is mediated via the regulation of the receptor tyrosine-kinase ligand EphrinA3 [37]. Animal experiments demonstrated that miR-210 was elevated after one day of MCAO and gradually decreased after 7 and 14 days of MCAO [38]. miR-424 promotes angiogenesis by inhibiting cullin 2 (CUL2), thereby increasing HIF-1*α* levels [39]. Recently, the miR-23-27-24 cluster has also been reported to have a prominent role in angiogenesis [40]. miR-378 promotes angiogenesis by targeting tumor suppressor candidate 2 (Fus-1) and suppressor of fused (Sufu), thus inducing indirect upregulation of VEGF and angiopoietin-1/2 [41].

### 2.4. Remyelination

In recent years, specific miRNAs such as miR-219, miR-138, miR-9, miR-23, and miR-19b have been found to participate in the regulation of oligodendrocyte differentiation and myelin maintenance, as well as in the pathogenesis of demyelination-related diseases (e.g., multiple sclerosis, ischemic stroke, and leukodystrophy) [42]. miR-19b, especially, plays essential roles in increasing the number of oligodendroglial cells [43]. The overexpression of miR-19b downregulates PTEN protein levels in Oligodendrocyte precursor cells (OPCs) by activating its downstream targets of the Akt signaling (PI3sOPCs) and the Akt signaling (PI3K/Akt/mTOR) pathway [44]. miR-19b increases the phosphorylation of Akt, but it does not affect its overall levels. The Akt1/2 kinase inhibitor cancels miR-19b-mediated OPC proliferation [43]. miR-145, -132, -200, and -182 are critical in the pathogenesis of ischemic stroke. The antagomir-mediated prevention of significantly upregulated miR-145 expression has been found to lead to an increased protein expression of its downstream target, superoxide dismutase-2 (SOD2), in the postischemic brain [45]. miR-132 regulates MeCP2 (methyl-CpG binding protein 2, also expressed in glial cells) expression, which is decreased in the preconditioned cortex. The down-regulation of miR-132 induces a rapid increase in the MeCP2 protein levels, but not the mRNA levels, in the mouse cortex [42]. The early activation of miR-200 family members improved neural cell survival via PHD2 mRNA silencing and subsequent HIF-1*α* (hypoxia-inducible factors-1*α*, a well-established transcription factor rapidly induced by hypoxia) stabilization [42].

## 3. miRNAs as Biomarkers of Cerebral Ischemia

Serum/plasma miRNAs derived from various tissues/organs are stable and resistant to nuclease digestion as well as other harsh conditions, including boiling, low/high pH, extended storage, freeze-thaw cycles [46]. Expression levels of miRNAs in blood have been found to be reproducible and indicative of the disease state [46]. Furthermore, miRNAs also exist in other body fluids, including urine, tear, ascetic fluid, and amniotic fluid [46]. 20 and 25 miRNA transcripts were detected in the blood of MCAO rats reperfused for 24 and 48 hours, respectively. Transcripts that were common to both the blood and brain at 24-hour reperfusion included rno-miR-16, -23a, -103, -107, -150, -185, -191, -292-5p, -320, -451, -494, and let-7 (a, d, f, and i). miRNAs found at 48-hour reperfusion in both the blood and brain were miR-26a, -26b, -103, -107, -140*, -150, -185, -195, -191, -214, -320, -328, -352, -494, and let-7 (a, c, and i) [2]. 

The correlation between blood and brain miR-210 in ischemic mice was positive. Compared to healthy controls, blood miRNA-210 was significantly decreased in stroke patients, especially at 7 days and 14 days of stroke onset. The cut off point of miR-210 in diagnosis was 0.505 with 88.3% sensitivity. MiR-210 level in stroke patients with good outcomes was significantly higher than patients with poor outcomes. Therefore blood miR-210 is a novel sensitive biomarker for clinical diagnosis and prognosis in acute cerebral ischemia [38]. miRNA profile of small artery (SA) stroke peripheral blood samples showed a distinctly different pattern from that of the large artery (LA) stroke samples [28]. Hence, the subtypes of stroke could be predicted using the microRNA profiling. miR-320 has been observed to be marginally down-regulated in all stroke patients with especially good outcome. The down-regulation of miR-320 could also lead to antiapoptotic processes [47] that could be useful in the restoration of normal cell or endovascular activities. Consequently it could be predictive of a favorable outcome via activation of angiogenesis in stroke patients [28].

### 3.1. miRNAs as Emerging Therapeutic Targets in Ischemic Diseases

miRNAs have been investigated as mediators of ischemic tissue damage. miR-200 family (miR-200a, miR-200b, miR-200c, miR-141, and miR-429) and miR-182 family (miR-182, miR-183, and miR-96) were upregulated early after ischemic preconditioning. Among them miR-200b, miR-200c, and miR-429 targeted PHD2 and had the best neuroprotective effect [48]. In the ischemia of cardiac myocytes, miR-199a was acutely downregulated as early as 30 minutes after ischemia, leading to rapid upregulation of its target HIF-1*α* [49]. Angiopoietin-1 is a vascular strengthening factor during vascular development and a protective factor for pathological vascular inflammation and leakage. The TT genotype (rs2507800) in the 3′-UTR of angiopoietin-1 may reduce the risk of stroke by interfering with miR-211 binding [50].

### 3.2. Clinical Implications and Problems

With the growing evidence for the involvement and the regulatory function of miRNAs in many pathophysiological processes, these small regulatory RNAs are evolving as promising therapeutic targets [51]. Whereas miRNAs as an treatment application in cerebrovascular diseases remain experimental so far and several limitations need to be solved before clinical practice.

### 3.3. miRNA-Replacement Therapy

After passing the cellular membrane, miRNA-mimicking oligonucleotides (miR-mimics) need to be integrated into the RNA-induced silencing complex [52] and induce translational inhibition of the degradation of their mRNA targets. Hence, possible chemical modifications that can increase the resistance to degradation of these oligonucleotide chemistries or facilitate their cellular uptakes are limited. Although overexpression of miRNAs by pre-miR-oligonucleotides or miR-mimics is a well-established method for the characterization of miRNA-function in vitro, only a few successful applications for the in vivo treatment of mammals exist so far [51].

### 3.4. miRNA Inhibition

AntagomiRs are single-stranded RNA molecules, modified by 2′-O-methyl and phosphorothioate substitution for stability. To enable cellular uptake, they are conjugated with cholesterols. These compounds can achieve significant miRNA knockdown [53] and have been used successfully to treat experimentally induced diseases in different organs and tissues [54, 55]. Clinical trials have successfully tested LNA-based drugs for the treatment of hepatitis C [56, 57] and several other LNA-based therapeutics are under development [58]. 

## 4. Future Prospects

**Figure 1:**
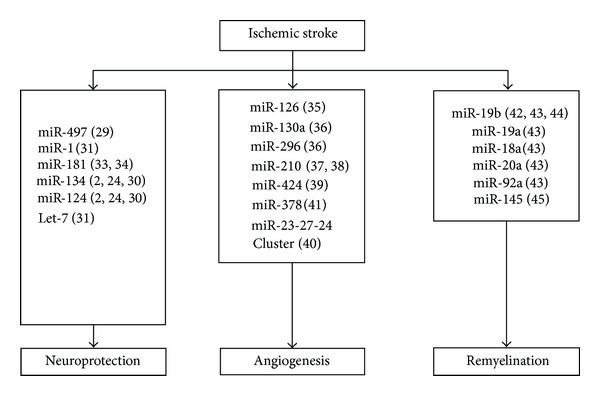
Regulation of miRNAs in ischemic stroke. Bracket indicates reference cited.

Identification of specific miRNAs as key regulators of the response to ischemia has opened new clinical avenues.  Figure 1 summarized the function of microRNAs in cerebral ischemia. Circulation miRNAs may be qualified as excellent non-invasive clinical biomarkers. During pathological processes, the expression of miRNAs is different in various cell types. Individual miRNAs can regulate the expression of multiple target genes, and manipulating miRNAs expression can influence an entire gene network and thereby modify complex disease pathologies [59]. Moreover, innovative strategies targeting miRNAs have been developed and could be applied in the treatment of ischemic diseases. Further studies on miRNAs are expected to shed new light in stroke therapy and management in the future.
